# Data needs for sea otter bioenergetics modeling

**DOI:** 10.1093/conphys/coae067

**Published:** 2024-10-08

**Authors:** Blaine D Griffen, Lexanne Klimes, Laura S Fletcher, Nicole M Thometz

**Affiliations:** Department of Biology, Brigham Young University, Provo, UT 84602, USA; Department of Biology, Brigham Young University, Provo, UT 84602, USA; Department of Biology, Brigham Young University, Provo, UT 84602, USA; Department of Biology, University of San Francisco, 2130 Fulton Street, San Francisco, CA 94117, USA

**Keywords:** Activity budgets, Body condition, Energy expenditure, Energy intake, *Enhydra lutris*, Foraging behavior, Metabolism, Model development

## Abstract

Sea otters are keystone predators whose recovery and expansion from historical exploitation throughout their range can serve to enhance local biodiversity, promote community stability, and buffer against habitat loss in nearshore marine systems. Bioenergetics models have become a useful tool in conservation and management efforts of marine mammals generally, yet no bioenergetics model exists for sea otters. Previous research provides abundant data that can be used to develop bioenergetics models for this species, yet important data gaps remain. Here we review the available data that could inform a bioenergetics model, and point to specific open questions that could be answered to more fully inform such an effort. These data gaps include quantifying energy intake through foraging by females with different aged pups in different quality habitats, the influence of body size on energy intake through foraging, and determining the level of fat storage that is possible in sea otters of different body sizes. The more completely we fill these data gaps, the more confidence we can have in the results and predictions produced by future bioenergetics modeling efforts for this species.

## Introduction

The survival and fitness of all organisms depends on their ability to maintain positive energy balance by effectively managing energy gains, losses, and allocation at appropriate timescales throughout their lifetime. Bioenergetics modeling is a key tool used to study and understand these dynamic processes, and such methods have long been used to examine energy balance in marine mammals. Several recent papers have reviewed bioenergetics modeling in marine mammals (Pirotta, 2002), provided guidance for estimating key parameters (Booth *et al*., 2003), reviewed available metabolic rates (Noren and Rosen, 2023) and growth patterns (Adamczak *et al*., 2023), and identified pressing data needs for improving our understanding of marine mammal bioenergetics and bioenergetic modeling (McHuron *et al*., 2022). These and other recent papers outline a rich history in, and promising future for marine mammal bioenergetics research. However, sea otters have either not been included or were minimally considered in these previous studies.

As the smallest marine mammal, balancing bioenergetic priorities is especially important for sea otters and must be done on much smaller time scales than other marine mammals. Due to their small body size and aquatic lifestyle, it has been proposed that sea otters have one of the highest mass-specific metabolic rates of any marine mammal (Yeates *et al*., 2007; Thometz *et al*., 2014; Noren and Rosen, 2023). Their relatively high metabolism means that adult sea otters must consume roughly one quarter of their body weight in food daily (Costa and Kooyman, 1982), resulting in rapid depletion of local prey resources as population densities increase (Tinker *et al*., 2008, 2012). Reduced abundance of prey results in otters spending more time foraging as prey quality and quantity decline and thus less time performing other essential activities (Estes *et al*., 1986; Walker *et al*., 2008; Thometz *et al*., 2016a). Additionally, foraging is an energetically expensive activity for otters due to high positive buoyancy resulting from large lungs and air trapped in fur (Fish *et al*., 2002; Yeates *et al*., 2007; Thometz *et al*., 2015). Thus, any increase in time spent foraging to maintain energy balance will reduce the amount of time and energy available for other essential behaviors and physiological processes such as resting, grooming, body maintenance and reproduction (Yeates *et al*., 2007; Thometz *et al*., 2016b).

For reproductive females, costs associated with gestation, lactation, and pup care are superimposed on already high baseline energy demands, resulting in intense energetic strain for female sea otters relative to other marine mammal species (Thometz *et al*., 2014; Thometz *et al*., 2016b). The uniquely high energetic burden of reproduction for female sea otters is amplified by the fact that they typically give birth to a pup every year from the time they reach sexual maturity until death (Gerber *et al*., 2004); essentially undergoing a continual cycle of gestation and lactation periods, each lasting approximately 6 months (Jameson and Johnson, 1993). At birth, sea otter pups are highly altricial and maintain high energy demands throughout dependency (Staedler, 2011; Thometz *et al*., 2014). Consequently, females must increase energy intake over the course of lactation to meet the growing energy needs of a pup as well as their own energy requirements, leading to greater energetic strain on females as pups mature (Cortez *et al*., 2016a, Thometz *et al*., 2016b). In high-density populations, the combined effects of decreased prey availability (Tinker *et al*., 2008, 2012) and high reproductive costs can lead to severe negative energy balance, resulting in the death of adult females at the end of lactation, a condition known as end lactation syndrome (Chinn *et al*., 2016, Miller *et al*., 2020). Within and across individuals, there is considerable variation in the duration of pup care (19-26 weeks, Jameson and Johnson, 1993) with clear trade-offs associated with early versus late weaning strategies. The longer a mother keeps a pup, the more likely she is to develop end lactation syndrome (Chinn *et al*., 2016); however, longer dependency periods also increase the likelihood of pup survival post-weaning (Jameson and Johnson, 1993, Riedman *et al*., 1994; Estes *et al*., 2003; Tinker *et al*., 2017). Thus, the decision of when to wean a pup can have important implications in terms of current and future reproductive success.

These behavioral decisions and associated trade-offs are all dependent on demographic-specific, individual energetics, but also have population level consequences, highlighting the importance of a bioenergetics perspective for this threatened species. Reduced to <2,000 individuals throughout their range during the fur trade of the 18^th^ and 19^th^ centuries, sea otter numbers have rebounded to ~150,000 today (Davis *et al*., 2019). Yet sea otters are still absent from much of their historic range (Davis *et al*., 2019) and continue to face threats from human impacts that result from their proximity to shore (Estes *et al*., 2003; Barrett, 2019), impacts from predators (Fomin *et al*., 2023) due to shifting food webs (Estes *et al*., 1998), increasing incidence of white shark related mortality (Tinker *et al*., 2016; Moxley *et al*., 2019), loss of prey and/or preferred habitat (McPherson *et al*., 2021) and resulting nutritional stress (Kuker and Barret-Lennard, 2010), increased incidence of disease (Miller *et al*., 2023), and contaminant exposure (Estes *et al*., 1997). Given their role as a keystone predator, the recovery and expansion of sea otters into their former range is broadly important as it can serve to enhance local biodiversity, promote community stability, and buffer against habitat loss in nearshore marine systems (Estes and Palmisano, 1974; Hughes *et al*., 2013, 2016; Smith *et al*., 2021; Nicholson *et al*., 2024). Bioenergetics models can be useful to inform conservation and/or management efforts.

Bioenergetics models have recently played a key role in conservation efforts for other marine mammals. For instance, bioenergetics models were combined with movement models to reveal the population consequences of disturbance from human activities that alter the behavior of harbor porpoises, gray seals, and harbor seals (Chudzińska *et al*., 2024), and similarly with beluga whales (John *et al*., 2024) and beaked whales (Hin *et al*., 2023). Bioenergetics models have also been used to explore the response of sperm whales to changes in prey availability (Silva *et al*., 2024). Bioenergetics models could similarly be used to enhance conservation efforts in sea otters. For instance, such models could be useful in identifying areas for reintroduction, or for determining the best locations for releasing juveniles that have been raised in captivity by surrogate mothers (Nicholson *et al*., 2023). Additionally, bioenergetics models could be used to predict the consequences of shifts in sea otter prey communities that accompany the loss of kelp beds along the Pacific coast (Smith *et al*., 2024). Despite these potential benefits of a bioenergetics model for sea otters, one has not yet been developed for this species. Although sea otters may appear to be a data-rich species relative to other marine mammals, several key data gaps remain, and filling these gaps would help to inform a robust bioenergetics model. Here we review the available data and point to specific open questions that could be answered to enhance the success of bioenergetics modeling efforts for sea otters.

## Existing Bioenergetics Data on Sea Otters

A general form for most bioenergetics models, following Booth *et al.* (2003), is:


(eq. 1) \begin{equation*} C=F+U+M+G+R+S \end{equation*}

Where *C* is the amount of energy consumed and is a function of foraging effort, prey availability, foraging success, and prey energy content. Some of this consumed energy is digested, while the remainder is lost during post-consumptive processing as feces (*F*). During the digestion process, energy is further lost to urine production (*U*). Energy that is not lost to feces or urine production is available to metabolize. This energy is used in metabolic maintenance costs (*M*), which are a function of activity (Yeates *et al*., 2007), presumably general body condition (though more data are needed to address this question in marine mammals, McHuron *et al*., 2022), and the amount and type of food being digested (i.e., the heat increment of feeding). Thus, metabolic maintenance costs are not independent from energy consumption. Energy not expended via metabolic costs is then available for production, either as growth of somatic tissue (*G*), reproduction (*R*), or energy storage, usually as glycogen, fat, or blubber (*S*). Thus, bioenergetics models must quantify all but one of these variables in order to reliably estimate the other using a mass or energy balance approach. Below, we summarize the information available for sea otters for each aspect of this general bioenergetics model.

### Consumption

Numerous studies have quantified energy intake during foraging. This has been done primarily using either shore-based observations with spotting scopes (Ostfeld, 1982; Garshelis *et al*., 1986; Hale *et al*., 2019; Tinker *et al*., 2019; Yee *et al*., 2020; LaRoche *et al*., 2023; Yee *et al*., 2023; Law *et al*., 2024) or using boat-based focal follow studies (Lee *et al*., 2010; Cortez *et al*., 2016a), although it is not clear that these methods are comparable, as focal follows could potentially alter foraging behavior of animals under observation. The majority of studies identify captured prey species to the lowest possible taxonomic level and approximate the size of the prey based on its size relative to the otter’s paw. This information is then used to calculate energy intake based on the energy density of different prey types available in the literature (usually from Oftedal *et al*., 2007) or measured within the same study (e.g., Garshelis *et al*., 1986; LaRoche *et al*., 2023). Many of these studies assume that energy densities taken from the literature apply to prey captured at the study site and at the season captured, which may not be the case given variation in energy density with location and season, as has been shown for the prey of other marine mammals (Mr̊rtensson *et al*., 1996; Logerwell and Schaufler, 2005; Spitz *et al*., 2018; IJsseldijk *et al*., 2021). In addition, studies often assume that captured prey are consumed with 100% efficiency, which may not be the case for several possible reasons, such as if some prey tissue is lost due to messy eating as observed in other aquatic predators (e.g., Dagg, 1974), loss in rough seas, or personal preference of the individual otter for specific tissues.

With these uncertainties in mind, energy intake rates of otters foraging in the field vary considerably across studies, ranging from 5-23 kcal/min (Table 1). Much of this variation may be explained by population-level and demographic differences between otters in different studies. For instance, there are consistent reductions in energy intake rates the longer that otters have occupied an area (Laidre and Jameson, 2006; Tinker *et al*., 2008, LaRoche *et al*., 2023), and as otter density increases (Hale *et al*., 2019; Tinker *et al*., 2019). Further, in studies that examined both males vs. females, females often consumed energy at a 1-2 kcal/min faster than males (Lee *et al*., 2010; Tinker *et al*., 2019), though Yee *et al*. (2020) found the opposite. Differences in energy intake between males and females are also influenced by tool use (Law *et al*., 2024). Additionally, foraging females that have small pups brought in 32% (Lee *et al*., 2010) and 44% (Bentall, 2005) less energy on average than females without pups foraging in the same area. Differences may also exist between northern and southern subspecies (Tinker *et al*., 2019), though observed differences may be explained by differences in time since establishment in the observed areas.

**Table 1:** Energy intake rates of otters reported in the literature, listed by location of study from north to south. Some values were converted from original units into kcal/min to facilitate direct comparisons. Energy intake rates were either provided directly in the text within original sources, or were captured from figures using Webplotdigitizer (standard errors were not able to be included when data were captured from certain plots).

Energy intake rate (kcal/min)	Subspecies (Location)	Sex	Methods	Situation	Reference
5.7 ± 0.8 (Long time) 11.06 ± 0.8 (short time)	Northern (Alaska)	Female	Shore-based	Time since colonization comparison	LaRoche *et al*., 2023
5.67 (Male) 7.34 (Lone female) 6.67 (Female with pup) 5.51 (Juvenile	Northern (Alaska)	Comparison	Focal follows (boat-based)	Comparison across demographics	Lee *et al*., 2010
5.07 (longest) 8.7 24.4 (shortest)	Northern (Prince William Sound)	Both	Shore-based	Comparison across 3 sites based on time since colonized	Garshelis *et al*., 1986
16.73 ± 2.21 (10.79-27.71)^*^	Northern (Washington)	Both	Shore-based	Across sites with different otter densities	Hale *et al*., 2019
16.05 ± 1.01 (Low density) 7.87 ± 0.44 (high density)	Northern & Southern (whole North American range)	Both	Shore-based	Comparison across population densities	Tinker *et al*., 2019
9.86 ± 0.22 (Females) 8.21 ± 2.41 (Males)	Northern & Southern (whole North American range)	Comparison	Shore-based	Comparison of males and females	Tinker *et al*., 2019
20.87 (2017) 17.55 (2018) 15.46 (2019)	Southern (San Nicolas Island)	Both	Shore-based	Comparison across years	Yee *et al*., 2020
20.88 (Winter) 22.60 (Spring) 13.57 (Summer) 11.25 (Fall)	Southern (San Nicolas Island)	Both	Shore-based	Comparison across seasons	Yee *et al*., 2020
18.28 (Female) 20.05 (Male) 13.38 (Unknown)	Southern (San Nicolas Island)	Comparison	Shore-based	Male vs. female comparison	Yee *et al*., 2020
10.83 (Female with large pup) 9.27 (Lone female)	Southern ( Piedras Blancas)	Female	Shore-based	Comparison of females with different aged pups at site with low prey abundance	Bentall, 2005
7.28 (Female with small pup) 12.88 (Female with large pup) 12.93 (Lone female)	Southern (San Nicholas Island)	Female	Shore-based	Comparison of females with different aged pups at site with high prey abundance	Bentall, 2005
8.60-9.79 (Female, 1-24% tool use) 8.60-6.21 (Female >24% tool use) 9.79-11.70 (Male 1-17% tool use) 9.79-5.02 (Male >17% tool use)	Southern (Big Sur, Monterey, San Luis Obispo, Piedras Blancas, Elkhorn Slough)	Both	Shore-based	Comparison with degree of tool use	Law *et al*., 2024

^*^Removed outlier site that the authors of the study also removed from analysis

### Pup self-provisioning (an aspect of consumption)

A large part of the energy budget of adult female otters is devoted to provisioning for pups. A female with a pup must forage to meet both her own needs and those of her pup (Thometz *et al*., 2016a). As stated in the previous section, foraging females with pups generally bring in less energy on average than females without pups foraging in the same area (Lee *et al*., 2010). Yet, this study did not document the age of pups, and foraging efforts by females generally increase with pup age in order to meet increasing energy demands (Thometz *et al*., 2016b). A single study has quantified foraging energy intake for female otters with pups of different ages. Bentall (2005) provides data on foraging energy intake for a lone female, a female with a small pup, and a female with a large pup foraging at San Nicolas Island in California’s Channel Islands, a site with low sea otter density. Mean energy intake by lone females and females with large pups were identical (12.9 kJ min^-1^), while energy intake was lower for females with small pups (7.3 kJ min^-1^); but, it should be noted that these data come from just three individual otters. However, if a bioenergetics model of adult females is accounting for energy provisioned to the pup, then this model also must account for energy brought in directly by the pup, since this too contributes to the overall mother-pup energy acquisition. Around day 63, pups begin to forage for themselves and are able to increasingly provision their own energetic needs until they are eventually weaned (Payne and Jameson, 1984; Staedler, 2011). We are not aware of any studies that have quantified pup prey capture while pups are still dependent on their mothers. However, previous studies have examined prey capture success during foraging dives by juveniles and subadults. For instance, Lee *et al*. (2010) found that juvenile otters (identified by smaller size and darker pelage, but age not determined) in Alaska brought in much less energy per dive on average (53.5 kJ) compared to all adult demographics (>75 kJ). Similarly, Laidre and Jameson (2006) observed otters in Washington state and found that prey capture success was lower in subadults (again, age not determined; 63%) than in adults (82%).

### Post-consumption processing (feces, urine, and heat increment of feeding)

Consumed energy that is lost to feces (incomplete digestion) and urine production, as well as to the heat increment of feeding have all been measured in sea otters ( Costa, 1982). These variables were measured in four captive sea otters that were either held long-term at SeaWorld, San Diego, CA, or were captured and held short-term while assimilation efficiency was measured. Each was fed a different diet of clam, abalone, or squid, and feces were collected, weighed, and the energy content was determined using bomb calorimetry. Fecal energy content was then compared to the energy content of the food consumed. On average, these otters assimilated 82% of the energy ingested, a relatively low value compared to values ≥90% commonly seen in other carnivores (e.g., Miller, 1978; Fadely *et al*., 1990). This low value can likely be attributed to the high rate of food consumption in otters (Costa, 1982), since assimilation efficiency often decreases with the amount of food consumed (e.g., Trumble *et al*., 2003). This same study also measured urine production in these captive otters, as well as in other captive and wild otters, and found that urine production accounts for an additional energy loss of 10% of ingested energy (Costa, 1982). In a separate study, Costa and Kooyman (1984) measured metabolic energy expenditure of postprandial otters compared to unfed otters and found that energy lost to the heat increment of feeding was equivalent to 10%-13% of ingested energy. Thus, accounting for all three of these processes leaves approximately 59%-62% of consumed energy that is available metabolically, after post-consumptive processing, to meet daily energetic needs. While the data for post-consumptive processing come from a limited number of observations, they are consistent with expected trends and more effort is therefore likely not necessary in this area.

### Activity budgets

The energy intake rates summarized above and in Table 1 must be combined with estimates of time spent foraging to yield overall energy intake on a daily basis. At the same time, metabolic energy expenditure varies with activity (see below). Thus, activity budgets provide a functional link between energy intake and energy expenditure. Activity budgets of sea otters have been abundantly estimated in studies conducted in numerous locations and under a wide range of conditions (summarized in Table 2). Activity has been estimated using various methods, including direct observations, either from shore or from a boat using focal follows, or using radio-telemetry and/or time-depth recorders (Davis and Bodkin, 2021); although previous work has challenged the relative accuracy of these different approaches (Garshelis *et al*., 1990). Several general patterns have emerged. For instance, adult females generally decrease foraging effort shortly after parturition in order to protect and care for their new pup (Sherrod *et al*., 1975; Esslinger *et al*., 2014; Gelatt *et al*., 2002, Cortez *et al.* 2016b, Thometz *et al*., 2016b), but progressively increase foraging effort as pups get older in order to meet increasing energetic demands of lactation and pup provisioning (Gelatt *et al*., 2002; Osterrieder and Davis, 2009; Cortez *et al*., 2016b; Thometz *et al*., 2016b; Tinker *et al*., 2019). In addition, individuals increase foraging effort as population density increases to compensate for reduced rates of energy intake as food becomes depleted (Estes *et al*., 1982; Thometz *et al*., 2016b; Tinker *et al*., 2008, 2019, and reviewed in Davis and Bodkin, 2021). Foraging effort also differs based on prey species selected (e.g., foraging effort increased when clams and urchins were consumed compared to when crabs or mussels were consumed, Kvitek *et al*., 1993) and prey species available (e.g., otters in Prince William Sound, AK spent more time foraging as large crabs became depleted, Garshelis *et al*., 1986). As noted above, pups increase foraging behavior as they age (Osterrieder and Davis, 2011), with independent juvenile females displaying some of the highest foraging effort of any demographic group (50% of time budget spent foraging) (Ralls and Siniff, 1990). This value is similar to the amount time spent foraging by females with large pups in both resource-abundant (Staedler, 2011: 50%; Thometz *et al*., 2016b: 47.7%) and resource-limited habitats (Thometz *et al*., 2016b: 48.2%), suggesting that ~50% time allocation to foraging may define a behavioral and/or physiological limit for this species.

**Table 2:** Activity budgets of sea otters measured in different studies. Numbers are percentage of time. Abbreviations in Comparison column are state abbreviations, where AK is Alaska, CA is California, WA is Washington. Only values for adult activity budgets are given, even when studies provide data on pup activity budgets (Walker *et al*., 2008; Cortez *et al*., 2016b). Empty cells indicate data for that behavior were not specifically collected. This behavior would therefore fall under the “Other” category in these instances.

Resting	Swimming	Foraging	Grooming	Other	Comparison	Reference
Area1: 61.8 Area2: 72.1 Area3: 53.4 Area4: 55.5	Area1: 9.6 Area2: 3.4 Area3: 8.0 Area4: 5.6	Area1: 6.7 Area2: 3.1 Area3: 12.6 Area4: 11.7	Area1: 19.7 Area2: 20.1 Area3: 23.5 Area4: 24	Area1: 2.2 Area2: 1.3 Area3: 2.5 Area4: 3.2	Across 4 different areas for adults in WA	Walker *et al*., 2008
<4 wks: 50 4<8 wks: 50 8<12 wks: 46	<4 wks: 21 4<8 wks: 16 8<12 wks: 8	<4 wks: 9 4<8 wks: 13 8<12 wks: 32	<4 wks: 2+ 4<8 wks: 28 8<12 wks: 9	<4 wks: 0 4<8 wks: 1 8<12 wks: 5	Females with different aged pups in AK	Cortez *et al*., 2016b
Boat: 50 Shore: 42	Boat: 14 Shore: 20	Boat: 19 Shore: 19	Boat: 16 Shore: 19	Boat: 2 Shore: 0	Boat-based vs. shore-based measurements in AK	Cortez *et al*., 2016b
42	15	18	15	10	Adult female with dependent pup in AK	Wolt *et al*., 2014
40.2	8.5	36.3	9.1	7.3	Adult males in CA	Yeates *et al*., 2007
Area 1: 53 Area 2: 57 Area 3: 63 Area 4: 62		Area 1: 26 Area 2: 21 Area 3: 28 Area 4: 23		Area 1: 22 Area 2: 22 Area 3: 9 Area 4: 15	Different areas in CA	Estes *et al*., 1986
Males: 50 Lone females: 50 Females with pups: 43	Males: 4 Lone females: 3 Females with pups: 3	Males: 47 Lone females: 47 Females with pups: 53			Male, females, females with pups in AK	Garshelis *et al.* 1986
49		38		13	Declining population in AK	Gelatt *et al*., 2002
Male: 46 Lone Female: 46 Female with pup: 45		Male: 40 Lone Female: 40 Female with pup: 40		Male: 14 Lone Female: 14 Female with pup: 15	Male vs female with or without pup in CA	Ralls and Siniff, 1990
Lone female: 46.7 Female with pup: 44.1	Lone female: 9.3 Female with pup: 10.4	Lone female: 28.8 Female with pup: 22.7	Lone female: 11.9 Female with pup: 17.9	Lone female: 3.3 Female with pup: 4.8	Lone females vs. females with pups in AK	Estes *et al*., 1982
Males: 46 Females: 48	Males: 5 Females: 10	Males: 38 Females: 41			Males vs. females in AK	Esslinger *et al*., 2014
		Fall: 42(F); 38.2(M) Winter: 42.8(F); 39(M) Spring: 43.6(F); 39.7(M) Summer: 36.6(F); 32.7(M)			Males vs. females by season in AK	Esslinger *et al*., 2014
27	26	14	19	14	Adult male in AK	Finerty *et al*., 2009
Small: 93.4 Med: 82.1 Large: 52.8	Small: 0 Med: 6.5 Large: 14.1	Small: 0 Med: 1.3 Large: 17.2	Small: 0.6 Med: 1.4 Large: 4.3	Small: 7.6 Med: 11.4 Large: 13.9	Different sized pups in AK	Osterrieder and Davis, 2011

(Continued)

**Table 2:** Continued

Resting	Swimming	Foraging	Grooming	Other	Comparison	Reference
Small: 42 Med: 45 Large: 37.3	Small: 31.5 Med: 28.6 Large: 17.4	Small: 10.5 Med: 8.6 Large: 28.9	Small: 16.1 Med: 17.6 Large: 11.2	Small: 0.7 Med: 1.6 Large: 5.8	Females with different sized pups in AK	Osterrieder and Davis, 2011
Day: 55.6 Night: 45	Day: 14.4 Night: 11.5	Day: 16 Night: 22.4	Day: 6.6 Night: 8.2	Day: 7.8 Night: 11.8	Day vs night in CA	Wilkin, 2003
1994: 21.6 1995: 39.8	1994: 17.4 1995: 7.1	1994: 33.3 1995: 24.8	1994: 22.9 1995: 14.2	1994: 5.7 1995: 1.5	1994 vs 1995 in CA	Feinholz, 1998
		High resources Male: 39.6 Lone Female: 34.9 Female small pup: 16.6 Female med pup: 29.4 Female lg pup: 47.7 Low resources Male: 45.6 Lone Female: 41.6 Female small pup: 27.3 Female med pup: 39.3 Female lg pup: 48.2			Different demographics and resource abundance	Thometz *et al*., 2016b
		Male: 42.7 Female: 38.4			Male vs. Females in CA	Tinker *et al.* 2017
Lone: 50.3 Very sm pup: 68.2 Small pup: 52.3 Lg pup: 43.4		Lone: 41.1 Very sm pup: 24.3 Small pup: 39.9 Lg pup: 48.1		Lone: 8 Very sm pup: 7.5 Small pup: 7.9 Lg pup: 8.4	Adult female reproductive status in CA	Tinker *et al*., 2019
1986: 62.6 1987: 66.1		1986: 9.5 1987: 11.2		1986: 27.9 1987: 22.6	1986 vs. 1987 in WA	Bowlby *et al*., 1988
		Low: 25 High: 40.8			Otter density in CA	Tinker *et al*., 2008
Male: 53 Female: 52		Male: 30 Female: 40		Male: 17 Female: 8	Male vs. female in AK	Bodkin *et al*., 2007
18	9	30	15	28	Territorial males in AK	Pearson and Davis, 2005

### Metabolic rates

In contrast to the numerous studies that have measured some aspect of energy intake in sea otters, fewer have measured energy expenditure (Morrison *et al.* 1974, Costa and Kooyman 1982, 1984; Williams, 1989; Yeates *et al*., 2007; Thometz *et al*., 2014, 2016a). Each of these studies measured metabolic rate on a relatively small number of individuals that differed in size, demographics, and behavior during measurements (e.g. resting, grooming, diving, foraging, etc.). Metabolic rates are known to differ broadly across conspecifics of a wide range of organism types (Burton *et al*., 2011; Konarzewski and Książek, 2013; Metcalfe *et al*., 2023), with important fitness implications (Burton *et al*., 2011). Despite this, metabolic rates of sea otters measured across these different studies, using different techniques, have been relatively consistent. Some general patterns emerge from these studies (summarized in Table 3), all of which are consistent with general metabolic patterns seen in other mammals. First, metabolism is generally lowest at rest, is higher for foraging (i.e., submerged swimming) and is generally highest for active grooming and surface swimming behaviors. Additionally, metabolic rates increase following stressful experiences (oil exposure) that reduce the insulative capacity of their fur, as well as during physiologically stressful periods (e.g. lactation and pup care). And finally, metabolic energy expenditure is higher on a mass-specific basis for pups and immature individuals than for adults.

**Table 3:** Metabolic rates of sea otters measured in different studies.

Metabolic rate (ml O_2_ min^-1^ kg^-1^)	Comparison made	Reference
Basal rates in air: 12 ± 1 ( -19 and 21°C) Basal rates in air: 11.17 (>21°C) Avg. rate in air: 16.5 Avg. rate in water: 20.17 Max rate during vigorous activity: 46.67	Changes in metabolism with air temperature; measurement also made in water and during strenuous activity (attacking cage); sex not reported	Morrison *et al*. 1974
Overall mean across temperatures: 11.7 ± 0.3 Post-oiling: 22.2 Post-washing (after oiling): 32.6	Changes in metabolism with temperature of adult females; impacts of oiling	Costa and Kooyman, 1982
Postprandial rate: ~17.5, decreasing to ~11 within 5 h of eating Control: 10-12	Changes in metabolism with time after feeding	Costa and Kooyman, 1984
Resting: 13.5 ± 1.81 Submerged swimming: 17.55 ± 1.70 Surface swimming: 29.61 ± 1.60	Different activities; adult males and females combined	Williams, 1989
Resting: 13.3 ± 0.9 Foraging: 21.6 ± 1.7 Grooming: 29.4 ± 2.6 Swimming: 29.6 ± 1.6 Other: 24.5	Different activities of adult males	Yeates *et al*., 2007
Resting in air Pre-molt pup: 23.18 ± 3.85 Molting pup: 22.16 ± 3.90 Post-molt pup: 17.49 ± 0.59 Moderately active in air Pre-molt pup: 31.91 ± 3.15 Molting pup: 28.29 ± 4.58 Post-molt pup: 21.00 ± 1.30 Highly active in air Pre-molt pup: - - Molting pup: 30.61 ± 2.26 Post-molt pup: 23.67 ± 1.04 Resting in water Pre-molt pup: 26.03 ± 3.34 Molting pup: 24.89 ± 7.98 Post-molt pup: 16.88 ± 1.75 Dependent immature: 16.08 ± 2.26 Juvenile: 13.63 ± 1.08 Moderately active in water Pre-molt pup: 31.36 ± 1.55 Molting pup: 32.55 ± 5.31 Post-molt pup: 23.43 ± 2.78 Dependent immature: 21.19 ± 4.24 Juvenile: 18.98 ± 2.28 Highly active in water Pre-molt pup: - - Molting pup: 35.38 ± 3.03 Post-molt pup: 30.82 ± 3.69 Dependent immature: 24.34 ± 5.76 Juvenile: 23.05 ± 3.74	Changes in metabolism of pups and juveniles with age and activity level and in or out of water	Thometz *et al*., 2014
Late gestation: 9.47 ± 0.16 Pup age 1-21 days: 11.37 ± 0.41 22-70 days: 11.38 ± 1.03 71-100 days: 14.04 ± 1.26 101-130 days: 17.23 ± 2.07 131-160 days: 15.62 ± 0.79 161-200 days: 15.26 ± 1.59 Nonreproductive: 11.35 ± 0.88	Resting metabolic rate of adult females at different reproductive stages	Thometz *et al*., 2016a

Activity-specific metabolic rate measurements can be combined with otter activity budgets to approximate demographic-specific field metabolic rates. Using this approach, the daily energetic demand of adult female sea otters (lone non-breeding) is estimated to be ~10 MJ d^-1^, but this estimate is doubled to ~20 MJ d^-1^ for reproductive females with a large pup (120-160 days old) that is still heavily dependent on its mother for nutrition (Thometz *et al*., 2016a). Similarly, the daily energetic demand for males has been variably estimated at 15.7 MJ d^-1^ (Yeates *et al*., 2007) and at 19.0 MJ d^-1^ (Finerty *et al*., 2009). For pups and juveniles, energetic demand increases with age and has been estimated at 2.3 MJ d^-1^ (pre-molt pups), 4.9 MJ d^-1^ (molting pups), 6.2 MJ d^-1^ (post-molt pups), 7.4 MJ d^-1^ (dependent immature individuals), and 8.6 MJ d^-1^ (juveniles) (Thometz *et al*., 2014). Given that metabolic costs are mass-specific, these daily costs would vary depending on the size of the otter, as described below.

Feeding studies with captive otters provide novel longitudinal data on daily energy intake rates for the same individuals over time (Hempstead and Larson, 2019; Iskandar *et al*., 2023). Although captive animals do not face the same energetic challenges associated with finding food, and likely also differ in daily activity budgets compared to otters in the field, data from these individuals can provide reasonable estimates of the lower bounds of energy needs within a model. Given this, long-term energy intake rates of captive otters would likely underestimate true field metabolic rates of wild sea otters if used in isolation.

### Body mass

As noted, the metabolic energy expenditures described above are mass-dependent, increasing as the size of the otter increases. Adult sea otters display a range of body masses. Northern sea otter pups grow at a faster rate (Riedman *et al*., 1994) and ultimately achieve larger body sizes as adults than their southern counterparts (Kenyon 1969, Davis and Lidicker, 1975). Yet even within a subpopulation, sea otters display a large range of adult body masses (southern subspecies females: 15-36 kg, males: 14-40 kg, Tinker *et al*., 2019). Thus, when modeling bioenergetics of sea otters, body mass must be explicitly included, since the greater than 2-fold difference in body mass of adult individuals will result in a greater than 2-fold difference between individuals in overall energy expenditure.

This relatively large range of body masses is surely influenced by body condition. However, body mass also increases with otter length (Rotterman and Monnett, 2002) as structural body mass increases, and there is considerable variation in body lengths of individual otters beyond three years of age (Tinker *et al*., 2019; Nicholson *et al*., 2020). This variation in body length may be determined by genetics and/or environmental factors, as otters at different sites have different growth rates based on age-to-length relationships (Tinker *et al*., 2019). Regardless of the driver, the result is that adult sea otters vary considerably in total body mass due to a combination of different structural and storage masses. In addition, these two factors likely interact, since longer otters have a larger frame that should both increase the structural body mass and simultaneously allow for more storage mass.

Even without differences in body condition, differences in otter length and resulting differences in structural body mass will itself yield large differences in metabolic energy expenditure. The California Department of Fish and Wildlife has collected sea otters stranded along the California coast since 1992. A portion of this database that they shared with us included 546 adult female otters stranded from 1992-2019. Within this dataset, tail-free body lengths ranged from 109 cm to 128 cm. Body mass also varied considerably at all lengths throughout this range. However, for non-pregnant adult females where no body fat was detected during necropsy, the lowest body mass (9.4 kg) was for an otter that was 109 cm long and the highest mass (26.4 kg) was for an otter that was 125 cm long. If we assume that both of these individuals follow the activity budget for a lone adult female in a resource rich environment in which females spend 59% of their time resting, 35% of their time foraging, and 6% of their time in other activities (Thometz *et al*., 2016b), then we can use the activity-specific mass-dependent metabolic costs of lone females (Thometz *et al*., 2016a) to compare the metabolic energy expenditure of these two individuals with very different structural body masses. This comparison reveals that the smaller of the two otters (9.4 kg) would expend 4.3 MJ per day during normal activity, while the larger of the two otters (26 kg) would expend 11.2 MJ per day while engaged in the same activities, a difference of 260%. This simple comparison highlights the importance of including structural body size when determining the energy expenditure of sea otters for use in bioenergetics models. Any additional difference in mass resulting from differential energy stores would further compound this difference.

## Remaining Data Needs

Two primary types of bioenergetics models have been applied to marine mammals, including accounting models used to examine energy balance, population energy requirements and overall prey consumption, and dynamic models used to predict the consequences of variable intake on energy reserves, survival, and reproductive output (Pirotta, 2002). Given the large variation in energy intake that has been reported, accounting model approaches could be used to examine specific populations in a specific location and time of year, but it is doubtful that these models would apply broadly across subspecies, sites or times. More general models used to inform across populations or seasons would therefore likely need to follow a dynamic approach, resulting in different questions that could be addressed. For instance, this approach could be used to make predictions about changes in activity budgets under different food intake rates, to assess different reproductive strategies (e.g., timing of pup weaning) as a function of maternal body condition, or to assess the viability of translocations or range expansion efforts. While considerable data are available for developing and informing such sea otter bioenergetics models, there are still several areas where additional data would be beneficial. Here we outline these data deficiencies and provide suggestions on how these data might be obtained.

### Energy intake rates

Although general foraging patterns and ranges of energy intake have been well defined across the sea otter range, energy intake rates are most often measured per minute of time spent foraging (kcal min^-1^), while the most useful metric for many bioenergetics modeling questions is daily energy intake (kcal day^-1^). Making this unit transition requires knowing the amount of time spent foraging each day, based on activity budgets. However, studies often measure one or the other of these metrics (foraging energy intake or activity budgets), but not both on the same individuals. There is even a lack of studies that measure both energy intake and activity budgets across different individuals that co-occur in the same area. Consequently, scaling rates of energy intake on a minute-by-minute basis to rates of energy intake per day requires the assumption that foraging energy intake data and activity budget data can be combined, even when collected on different individuals, often in different locations or at different times of the year. The high context-dependency of both energy intake rates and activity budgets measured previously calls this assumption into question. For instance, even when conducted at the same time but in different locations, consumption rates differ across locations with the length of time that otters have been in the area (e.g., Garshelis *et al*., 1986), and/or otter density (e.g., Hale *et al*., 2019), resulting in very different body conditions for otters across sites (Tinker *et al*., 2019). Similarly, foraging at the same location but at different times may also result in different rates of energy intake due to seasonal differences in prey energy content (LaRoche *et al*., 2023) or seasonal variation in foraging success (Kruuk and Moorhouse, 1990). Modelling efforts based on existing data would need to be careful to combine foraging and activity data only from the same region (i.e., habitats with the same prey availability) and season. Moving forward, the most useful empirical approach from an energy budget perspective is to collect data on rates of energy intake and activity budgets at the same site and at the same time, and ideally, on the same individuals.

Demographic differences likely account for some of the large amount of variation in energy intake that is reported in the literature, but this remains unclear because demographic data are not always included. Therefore, additional data are needed to quantify energy intake for at least four specific demographic groups, including in both prey-abundant and prey-limited habitats. We list these here in order of decreasing importance for bioenergetic modeling. First, based on data from just three otters, it appears that female energy intake is greatly affected by the age of a dependent pup (Bentall, 2005). Additional data are needed to clarify how these energy intake rates change as a function of pup age and to examine this relationship across habitats of different quality. Second, it would be useful to quantify foraging success of juveniles during the transition between weaning and adulthood, a period that is likely one of rapidly changing foraging capabilities. Two previous studies showed that prey capture success was lower for juveniles than for adults (Lee *et al*., 2010) and that overall energy intake was lower per dive for juveniles than for adults (Laidre and Jameson, 2006). Both of these studies were conducted on northern otters, and neither quantified juvenile age. It would be useful to know how foraging success varies with habitat quality and with juvenile age. Third, we have no data on energy intake of foraging pups of different ages. Pup foraging efforts begin around day 63 (Staedler, 2011) and increase gradually until weaning (Payne and Jameson, 1984), but it is unclear how much energy they obtain to supplement or replace energy acquired from the mother. However, given that mothers with older pups appear to maximize time spent foraging regardless of prey availability (Ralls and Siniff, 1990; Staedler, 2011; Thometz *et al*., 2016b), energy acquired directly by the pup may have limited impact on energy dynamics of the mother. Fourth, we lack data on how tooth wear may limit feeding capabilities of older otters (Winer *et al*., 2013; Nicholson *et al*., 2020), resulting in reduced feeding rates, foraging success, or shifts in diet type that may all influence energy intake rates. While these data are lacking, older individuals are at or near the end of reproductive life, and these data will therefore be less important to bioenergetic models. In general, quantifying energy intake for these different demographic groups would enable more situation-specific balancing of energy intake and expenditure, permitting useful energy budgets to be constructed at the individual level, where key parameters can be modeled to vary throughout life based on age, size, environmental context, etc.

A key focus of a bioenergetics approach, including bioenergetics modeling, is to understand how individual organisms balance the rates of energy intake and expenditure. As noted above, energy expenditures in adult sea otters are generally measured and expressed on a mass-specific basis, therefore implicitly accounting for the reality that larger individuals will have higher absolute metabolic costs in support of their larger bodies. In contrast to this, consumption rates of adults have been measured and mostly reported without concern for otter size. Given that larger individuals have higher energy expenditure, it should be expected that these same individuals will have higher rates of energy intake, but we lack empirical data to parameterize such a relationship for wild sea otters. Data are available to qualitatively examine this relationship in captive animals. Hempstead and Larson (2019) summarize data on 65 otters held in 24 zoos and aquaria globally. They state that body masses varied from 17 to 35 kg for otters from ages 1 to 21, and that the caloric intake varied from 96 to 215 kcal kg^-1^ d^-1^. However, while they clarify that the mass-specific food offered daily was negatively correlated with body mass (ranging from 12 to 26% of body mass by weight), they do not provide enough information to determine the relationship between body mass and absolute caloric intake. Iskandar *et al*. (2023) summarize energy intake by 10 sea otters held over three decades at the Vancouver, Canada Aquarium. They report that males were 25 to 42% larger than females and had a 24 to 58% higher caloric intake. They also report that mass-specific energy intake rates plateaued at ~650 kJ kg^-1^ d^-1^ for both males and females, suggesting that the relationship between body mass and energy intake is nonlinear. While these studies demonstrate higher energy intake for otters of larger body size, they do not provide sufficient information to quantify the relationship between body mass and energy intake. Understanding variation in energy intake with body size is important not only for balancing energy budgets at the individual level, but also for scaling up energy intake and impacts on prey abundance at the population level. Mismatches between size-dependent energy expenditure and size-dependent energy intake may be expected to reduce the success of larger individuals relative to smaller individuals in poor quality habitats. This could be easily examined with bioenergetics modeling, but would require data on size-dependent energy consumption.

Finally, the majority of published studies to date that examine sea otter foraging have calculated energy intake based on prey size and type using energy values measured by Oftedal *et al*. (2007), with the implicit assumption that these values applied to prey captured at the location and time of year in which the focal study was conducted. However, a recent study measured nutritional content of clams, crabs, sea cucumbers, snails, and urchins and found that there was considerable seasonal variation in energy, lipid, and protein content across these prey species (LaRoche *et al*., 2023). More studies are therefore needed that measure prey energy content at the locations and times where foraging is measured in order to more accurately estimate energy intake.

### Fat storage

While sea otters are the consummate income breeders, they do still experience a period of reduced foraging shortly after parturition in order to care for their pup and ensure its safety. Female sea otters are able to build limited fat stores during late gestation, presumably via reduced metabolic demands during that time (Thometz *et al*., 2016b), and it has been hypothesized that these fat stores play an important role energetically during the first weeks postpartum (Chinn *et al*. 2016). However, to date there is little quantitative information regarding the amount of fat that sea otters typically store. A single study reported that fat stores accounted for 1.8% of body mass for a single adult female otter during necropsy (Tarasoff, 1974). As discussed above, sea otters vary considerably in length and therefore in the size of their frame that can support fat storage. Thus, it is reasonable to assume that the mass of stored fat may vary across individuals of different length. This is especially important given that metabolism typically scales with mass to the 0.75 power (Kooyman, 1989), meaning that mass-specific maintenance costs should decrease as body mass increases, and fat stores typically scale 1:1 with body mass (Ryg *et al*., 1993; Prothero, 1995; Cornick *et al*., 2016; Antoł and Kozlowski, 2020). This could allow larger females to allocate more energy to fat storage during gestation in preparation for the initial weeks of pup care when foraging effort is reduced (Thometz *et al*., 2016b), and use those fat stores for longer periods. However, testing this prediction requires additional data on the scaling of metabolism and fat reserves in sea otters as a function of structural body mass.

The dataset described above from the California Department of Fish & Wildlife on stranded sea otters also included an estimate of stored fat (i.e., ‘fatscore’) that was observed during necropsy, and ranged from 0 (no observable fat) to 4 (abundant fat stores). Fat storage was common, with 165 of 546 otters (30.2%) having a fatscore of 3 or 4. Body masses ranged from 9.4 kg (109 cm length) for an otter with no fat to 32.1 kg (124 cm length) for an otter with abundant fat, while for a given length of otter, the difference between individuals with no fat and individuals with maximal fat often was ~10 kg. It is unclear how much of this difference in mass is attributable to fat storage rather than to differences in skeletal, muscle, or visceral mass. Thus, while this provides evidence that fat storage is common in sea otters, it does not enable a quantitative assessment of how much fat is stored, nor of the potential impact this fat storage may have on the energetics of different demographics in different locations or situations. In the absence of fat, females that cannot bring in enough energy through foraging will metabolize their lean tissue during advanced end lactation syndrome (Chinn *et al*. 2016). However, metabolism of fat and lean tissue provides very different amounts of energy (39.3 MJ kg^-1^ vs 18.0 MJ kg^-1^, respectively, Schmidt-Nielsen, 1997). Consequently, their metabolism has very different implications for animal energetics (Molnár *et al*. 2009). Additional clarity could be provided through quantitative empirical measurements of fat storage in sea otters of varying demographic groups and reproductive states. Body fat should also be examined as a function of body lengths, since longer otters have a larger frame and should therefore be expected to carry more body fat for a given body conditions score. Fat storage could be directly quantified through dissection and weighing as part of the necropsy procedure for stranded otters. Building a sufficient database on body fat to address the questions highlighted here would likely require considerable time, since it would rely on opportunistic necropsy of stranded otter carcasses. Alternatively (or in addition), relative body fat could be assessed on live animals using bioelectric impedance analysis (Kyle *et al*., 2004), a method that has favorably been used previously with polar bears (Pagano *et al*., 2017) and pinnipeds (Bowen *et al*., 1999). Ultimately, obtaining data on body fat storage is of high importance, especially for accurate dynamic bioenergetic models where the entire state space of stored energy is required. Predictions of energy-dependent behavior derived from dynamic models can vary drastically depending on estimates of energy storage and energy dynamics (Griffen *et al*., 2022).

### Less pressing data deficiencies

In addition to the relatively more important data needs addressed above, there are other areas where additional data could help hone bioenergetic models, but are likely less necessary. One of these is the influence of body composition on metabolic costs. As described above, metabolic energy expenditure has been measured for otters engaged in various activities, including foraging, resting, grooming, etc. These measurements, however, have not accounted for possible changes in metabolism that can occur due to changes in body composition. Work in other mammals shows that metabolic rate is depressed in starved animals that have lost a considerable amount of body fat and protein stores (Fuglei and Øritsland, 1999). And in marine mammals specifically, correlations between body composition and metabolic rate have also been observed (Sparling *et al*., 2006). Given the strong variation in body composition between healthy female sea otters and those experiencing end lactation syndrome (Chinn *et al*., 2016), it may be expected that metabolic rates will also differ as end lactation syndrome progresses. Understanding this variation would inform the time course of onset and progression of end lactation syndrome. This could be quantified by measuring resting metabolic rates of animals in rehabilitation programs that are at different health levels and then comparing this to healthy otters.

A recent survey found that estimating field metabolic rates was the number one thing needed for improving our understanding of marine mammal energetics in general (McHuron *et al*., 2022). Although a number of studies have estimated the field metabolic rate of sea otters of various demographics, these have used activity-specific energetic data collected with healthy, captive sea otters combined with activity budgets of wild individuals (Yeates *et al*., 2007; Thometz *et al*., 2014, 2016a), relied on modeling approaches (Cortez *et al*., 2016a), or used energy intake data from captive individuals (Iskandar *et al*., 2023). Thus, we lack direct measurements of field metabolic rate (FMR) for this species. Such direct measures could be made with free-ranging sea otters using the doubly labeled water technique, as has been successfully done with other marine mammals (Sparling *et al*. 2008), and could validate or refine previous estimates of FMR. If combined with a body condition score for each individual, this method could also improve our understanding of the impact of body condition on metabolism. Further, this type of study could reveal any metabolic differences between subspecies (see below). However, it may be difficult to successfully utilize this technique with sea otters given their high metabolic rates, the need for capturing and sampling the same individuals repeatedly over a short timeframe (e.g. days), and any site-specific constraints of capturing sea otters in the field. Ultimately, while field metabolic rates for sea otters have yet to be measured empirically, this may be difficult in practice and may yield fewer benefits for bioenergetics modeling than filling some of the data deficiencies on the energy income side of the equation discussed above.

### Differences between northern and southern otters

Finally, there may also be important differences between subspecies that should be acknowledged when developing bioenergetic models. While there is only a single species of sea otter (*Enhydra lutris*), there are three recognized subspecies - the Asian sea otter *Enhydra lutris lutris* that occurs in the western Pacific, the northern sea otter *Enhydra lutris kenyoni* that occurs in the eastern Pacific from Oregon to Alaska, and the southern sea otter *Enhydra lutris nereis* that occurs in central California in the Eastern Pacific (Campbell and Santana, 2017). The two subspecies in North America have two primary differences that are pertinent to bioenergetic modeling. First, northern sea otters achieve greater body size than southern sea otters (Kenyon, 1969). Second, northern sea otter pups exhibit faster growth rates than their southern counterparts (Riedman *et al*., 1994). These differences have bioenergetic implications for metabolic costs, energy intake requirements, the duration of pup care, and pup survival. It is unclear whether metabolic costs of reproductive females differ between northern and southern sea otters, whether the larger body size of northern otters imparts an advantage in energy storage prior to parturition, and whether the faster growth rates of northern sea otter pups impart an advantage to pup survival. We suggest that data used to inform individual modeling attempts should focus on a single subspecies rather than mixing data across subspecies.

## Conclusion

Because sea otter survival and reproductive success is determined largely by energy balance, we believe a bioenergetics approach would be useful in addressing many questions related to sea otter ecology and conservation. Facilitating such modeling efforts could be of broad interest, as sea otters are important keystone predators within nearshore marine ecosystems that enhance the biodiversity, stability, and resilience of the habitats in which they reside (Estes and Palmisano, 1974; Hughes *et al*., 2013, 2016; Smith *et al*., 2021). Thus, promoting the recovery and expansion of sea otters back into their historic range has the capacity to buffer against habitat loss and degradation of nearshore marine systems throughout the North Pacific (Nicholson *et al*., 2024). We have identified several knowledge gaps and areas where additional data could greatly facilitate a bioenergetics modeling approach for sea otters. Of particular importance are additional data on energy consumption by females with different age pups foraging in different quality habitats, data on energy consumption as a function of body size, on energy intake and activity budgets collected simultaneously on the same individuals, and on fat storage in otters of different body sizes. While energy accounting models may be profitably developed using existing data to examine energy balance in targeted situations, developing broader dynamic models to predict strategies under novel conditions requires data across a broader state space than currently exists. Filling these data needs is therefore necessary before these types of models can confidently be developed and used to inform conservation efforts.

## Data Availability

No data are associated with this manuscript.

## References

[ref1] Adamczak SK, McHuron EA, Christiansen F, Dunkin R, McMahon CR, Noren S, Costa DP (2023) Growth in marine mammals: a review of growth patterns, composition and energy investment. Conserv Physiol 11: coad035. 10.1093/conphys/coad035. 37492466 PMC10364341

[ref2] Antoł A, Kozłowski J (2020) Scaling of organ masses in mammals and birds: phylogenetic signal and implications for metabolic rate scaling. ZooKeys 982: 149–159. 10.3897/zookeys.982.55639. 33239956 PMC7652810

[ref3] Barrett HE (2019) The energetic cost of anthropogenic disturbance on the southern sea otter (Enhydra lutris nereis). San José State University.

[ref4] Bentall GB (2005) Morphological and behavioral correlates of population status in the southern sea otter, Enhydra lutris nereis: a comparative study between central California and San Nicolas Island (Doctoral dissertation. University of California, Santa Cruz).

[ref116] Bodkin JL, Monson DH, Esslinger GG (2007) Activity budgets derived from time-depth recorders in a diving mammal. J Wildl Manage 71: 2034–2044. 10.2193/2006-258.

[ref6] Booth CG, Guilpin M, Darias-O’Hara AK, Ransijn JM, Ryder M, Rosen D, Costa DP (2023) Estimating energetic intake for marine mammal bioenergetic models. Conserv Physiol 11: coac083. 10.1093/conphys/coac083. 36756464 PMC9900471

[ref7] Bowen WD, Beck CA, Iverson SJ (1999) Bioelectrical impedance analysis as a means of estimating total body water in grey seals. Can J Zool 77: 418–422. 10.1139/z98-223.

[ref8] Bowlby CE, Troutman BL, Jeffries S (1988) Sea otters in Washington: distribution, abundance, and activity patterns. National Coastal Resources Research & Development Institute, Newport OR.

[ref9] Burton T, Killen SS, Armstrong JD, Metcalfe NB (2011) What causes intraspecific variation in resting metabolic rate and what are its ecological consequences? Proc R Soc B 278: 3465–3473. 10.1098/rspb.2011.1778. PMC3189380 21957133

[ref10] Campbell KM, Santana SE (2017) Do differences in skull morphology and bite performance explain dietary specialization in sea otters? J Mammal 98: 1408–1416.

[ref5] Chinn SM, Miller MA, Tinker MT, Staedler MM, Batac FI, Dodd MM, Henkel LA (2016) The high cost of motherhood: end-lactation syndrome in southern sea otters (Enhydra lutris nereis) on the Central California Coast, USA. Journal of wildlife diseases 52: 307–318. 26967137 10.7589/2015-06-158

[ref11] Chudzińska M, Klementisová K, Booth C, Harwood J (2024) Combining bioenergetics and movement models to improve understanding of the population consequences of disturbance. Oikos 2024: e10123. 10.1111/oik.10123.

[ref12] Cornick LA, Quakenbush LT, Norman SA, Pasi C, Maslyk P, Burek KA, Hobbs RC (2016) Seasonal and developmental differences in blubber stores of beluga whales in Bristol Bay, Alaska using high-resolution ultrasound. J Mammal 97: 1238–1248. 10.1093/jmammal/gyw074. 29899579 PMC5993092

[ref13] Cortez M, Goertz CE, Gill VA, Davis RW (2016a) Development of an altricial mammal at sea: II. Energy budgets of female sea otters and their pups in Simpson Bay. Alaska. *J Exp Mar Biol Ecol* 481: 81–91. 10.1016/j.jembe.2016.03.018.

[ref14] Cortez M, Wolt R, Gelwick F, Osterrieder SK, Davis RW (2016b) Development of an altricial mammal at sea: I. Activity budgets of female sea otters and their pups in Simpson Bay. Alaska J Exp Mar Biol Ecol 481: 71–80. 10.1016/j.jembe.2015.05.020.

[ref15] Costa DP (1982) Energy, nitrogen, and electrolyte flux and sea water drinking in the sea otter *Enhydra lutris*. Physiol Zool 55: 35–44. 10.1086/physzool.55.1.30158441.

[ref111] Costa DP, Kooyman GL (1982) Oxygen consumption, thermoregulation, and the effect of fur oiling and washing on the sea otter, *Enhydra lutris*. Canadian Journal of Zoology 60: 2761–7.

[ref16] Costa DP, Kooyman GL (1984) Contribution of specific dynamic action to heat balance and thermoregulation in the sea otter *Enhydra lutris*. Physiol Zool 57: 199–203. 10.1086/physzool.57.2.30163705.

[ref17] Dagg MJ (1974) Loss of prey body contents during feeding by an aquatic predator. Ecology 55: 903–906. 10.2307/1934430.

[ref18] Davis RW, Bodkin JL (2021) Sea otter foraging behavior. In Ethology and Behavioral Ecology of Sea Otters and Polar Bears (pp. 57-81). Cham: Springer International Publishing, 10.1007/978-3-030-66796-2_4.

[ref19] Davis J, Lidicker WZ (1975) The taxonomic status of the southern sea otter. Proc California Acad Sci 40: 429–437.

[ref20] Davis RW, Bodkin JL, Coletti HA, Monson DH, Larson SE, Carswell LP, Nichol LM (2019) Future directions in sea otter research and management. Front Mar Sci 5: 411943. 10.3389/fmars.2018.00510.

[ref21] Esslinger GG, Bodkin JL, Breton AR, Burns JM, Monson DH (2014) Temporal patterns in the foraging behavior of sea otters in Alaska. J Wildl Manage 78: 689–700. 10.1002/jwmg.701.

[ref22] Estes JA, Palmisano JF (1974) Sea otters: their role in structuring nearshore communities. Science 185: 1058–1060. 10.1126/science.185.4156.1058. 17738247

[ref23] Estes JA, Jameson RJ, Rhode EB (1982) Activity and prey election in the sea otter: influence of population status on community structure. Am Nat 120: 242–258. 10.1086/283985.

[ref24] Estes JA, Underwood KE, Karmann MJ (1986) Activity-time budgets of sea otters in California. J Wildl Manage 50: 626–636. 10.2307/3800973.

[ref25] Estes JA, Bacon CE, Jarman WM, Norstrom RJ, Anthony RG, Miles AK (1997) Organochlorines in sea otters and bald eagles from the Aleutian archipelago. Mar Pollut Bull 34: 486–490. 10.1016/S0025-326X(96)00178-6.

[ref26] Estes, J. A., Tinker, M. T., Williams, T. M., & Doak, D. F. (1998). Killer whale predation on sea otters linking oceanic and nearshore ecosystems. Science, 282(5388), 473-476, 10.1126/science.282.5388.473. 9774274

[ref27] Estes J, Hatfield BB, Ralls K, Ames J (2003) Causes of mortality in California Sea otters during periods of population growth and decline. Mar Mamm Sci 19: 198–216. 10.1111/j.1748-7692.2003.tb01102.x.

[ref28] Fadely BS, Worthy GA, Costa DP (1990) Assimilation efficiency of northern fur seals determined using dietary manganese. J Wildl Manage 54: 246–251. 10.2307/3809037.

[ref29] Feinholz D (1998) Abundance, distribution, and behavior of the southern sea otter (*Enhydra lutris nereis*) in a California estuary. Aquat Mamm 24: 105–116.

[ref30] Finerty SE, Wolt RC, Davis RW (2009) Summer activity pattern and field metabolic rate of adult male sea otters (*Enhydra lutris*) in a soft sediment habitat in Alaska. J Exp Mar Biol Ecol 377: 36–42. 10.1016/j.jembe.2009.06.015.

[ref31] Fish FE, Smelstoys J, Baudinette RV, Reynolds PS (2002) Fur doesn't fly, it floats: buoyancy of pelage in semi-aquatic mammals. Aquat Mamm 28: 103–112.

[ref32] Fomin SV, Fedutin ID, Borisova EA, Meschersky IG, Filatova OA (2023) Sea otters (*Enhydra lutris*) found in the stomach of a stranded killer whale (*Orcinus orca*) in the Commander Islands, Western north pacific. Aquat Mamm 49: 462–467. 10.1578/AM.49.5.2023.462.

[ref33] Fuglei E, Øritsland NA (1999) Body composition, resting and running metabolic rates, and net cost of running in rats during starvation. Acta Physiol Scand 165: 203–210. 10.1046/j.1365-201x.1999.00511.x. 10090332

[ref34] Garshelis DL, Garshelis JA, Kimker AT (1986) Sea otter time budgets and prey relationships in Alaska. J Wild Manage 50: 637–647. 10.2307/3800974.

[ref35] Garshelis DL, Ames JA, Hardy RA, Wendell FE (1990) Indices used to assess status of sea otter populations: a comment. J Wildl Manage 54: 260–269. 10.2307/3809039.

[ref36] Gelatt TS, Siniff DB, Estes JA (2002) Activity patterns and time budgets of the declining sea otter population at Amchitka Island, Alaska. J Wildl Manage 66: 29–39. 10.2307/3802868.

[ref37] Gerber LR, Tinker MT, Doak DF, Estes JA, Jessup DA (2004) Mortality sensitivity in life-stage simulation analysis: a case study of southern sea otters. Ecol Appl 14: 1554–1565.

[ref38] Griffen BD, Whiteman JP, Pullan S (2022) Significance of autumn and winter food consumption for reproduction by southern Beaufort Sea polar bears, *Ursus maritimus*. Polar Biol 45: 1351–1362. 10.1007/s00300-022-03066-9.

[ref39] Hale JR, Laidre KL, Tinker MT, Jameson RJ, Jeffries SJ, Larson SE, Bodkin JL (2019) Influence of occupation history and habitat on Washington Sea otter diet. Mar Mamm Sci 35: 1369–1395. 10.1111/mms.12598.

[ref40] Hempstead C, Larson S (2019) Short note: sea otter (Enhydra lutris) diet diversity in zoos and aquariums. Aquat Mamm 45: 374–379. 10.1578/AM.45.4.2019.374.

[ref41] Hin V, De Roos AM, Benoit-Bird KJ, Claridge DE, DiMarzio N, Durban JW, Falcone EA, Jacobson EK, Jones-Todd CM, Pirotta E et al. (2023) Using individual-based bioenergetic models to predict the aggregate effects of disturbance on populations: a case study with beaked whales and navy sonar. PloS One 18: e0290819. 10.1371/journal.pone.0290819. 37651444 PMC10470956

[ref42] Hughes BB, Eby R, Van Dyke E, Tinker MT, Marks CI, Johnson KS, Wasson K (2013) Recovery of a top predator mediates negative eutrophic effects on seagrass. Proc Nat Acad Sci 110: 15313–15318. 10.1073/pnas.1302805110. 23983266 PMC3780875

[ref43] Hughes BB, Hammerstrom KK, Grant NE, Hoshijima U, Eby R, Wasson K (2016) Trophic cascades on the edge: fostering seagrass resilience via a novel pathway. Oecologia 182: 231–241. 10.1007/s00442-016-3652-z. 27167224

[ref44] IJsseldijk LL, Hessing S, Mairo A, Ten Doeschate MT, Treep J, van den Broek J, Leopold MF (2021) Nutritional status and prey energy density govern reproductive success in a small cetacean. Sci Rep 11: 19201. 10.1038/s41598-021-98629-x. 34725464 PMC8560860

[ref45] Iskandar S, Adelsheim J, Rosen DA (2023) The effects of age and sex on the energy intake of Captive Sea otters (Enhydra lutris): implications for captive management and species conservation. Aquat Mamm 49: 347–355. 10.1578/AM.49.4.2023.347.

[ref46] Jameson RJ, Johnson AM (1993) Reproductive characteristics of female sea otters. Mar Mamm Sci 9: 156–167. 10.1111/j.1748-7692.1993.tb00440.x.

[ref47] John JS, Christen DR, Flammer KL, Kendall TL, Nazario EC, Richter BP, Gill V, Williams TM (2024) Conservation energetics of beluga whales: using resting and swimming metabolism to understand threats to an endangered population. J Exp Biol 227: jeb246899. 10.1242/jeb.246899. 38483264 PMC11070638

[ref114] Kenyon KW (1969) The sea otter in the eastern Pacific Ocean. US Bureau of Sport Fisheries and Wildlife.

[ref48] Konarzewski M, Książek A (2013) Determinants of intra-specific variation in basal metabolic rate. J Comp Physiol B 183: 27–41. 10.1007/s00360-012-0698-z. 22847501 PMC3536993

[ref49] Kooyman GL (1989) Diverse Divers. Springer-Verlag, New York. 200 pages, 10.1007/978-3-642-83602-2

[ref50] Kruuk H, Moorhouse A (1990) Seasonal and spatial differences in food selection by otters (*Lutra lutra*) in Shetland. J Zool 221: 621–637. 10.1111/j.1469-7998.1990.tb04021.x.

[ref51] Kuker K, Barret-Lennard L (2010) A re-evaluation of the role of killer whales Orcinus orca in a population decline of sea otters Enhydra lutris in the Aleutian Islands and a review of alternative hypotheses. Mammal Rev 40: 103–124. 10.1111/j.1365-2907.2009.00156.x.

[ref52] Kvitek RG, Bowlby CE, Staedler M (1993) Diet and foraging behavior of sea otters in Southeast Alaska. Mar Mamm Sci 9: 168–181. 10.1111/j.1748-7692.1993.tb00441.x.

[ref53] Kyle UG, Bosaeus I, De Lorenzo AD, Deurenberg P, Elia M, Gómez JM, Composition of the ESPEN Working Group (2004) Bioelectrical impedance analysis—part I: review of principles and methods. Clin Nutr 23: 1226–1243. 10.1016/j.clnu.2004.06.004. 15380917

[ref54] Laidre KL, Jameson RJ (2006) Foraging patterns and prey selection in an increasing and expanding sea otter population. J Mammal 87: 799–807. 10.1644/05-MAMM-A-244R2.1.

[ref55] LaRoche NL, King SL, Fergusson EA, Eckert GL, Pearson HC (2023) Macronutrient composition of sea otter diet with respect to recolonization, life history, and season in southern Southeast Alaska. Ecol Evol 13: e10042. 10.1002/ece3.10042. 37153015 PMC10154889

[ref56] Law CJ, Tinker MT, Fujii JA, Nicholson T, Staedler M, Tomoleoni JA, Young C, Mehta RS (2024) Tool use increases mechanical foraging success and tooth health in southern sea otters (*Enhydra lutris nereis*). Science 384: 798–802. 10.1126/science.adj6608. 38753790

[ref57] Lee OA, Gelwick F, Davis RW (2010) Summer foraging tactics in sea otters (*Enhydra lutris*): maintaining foraging efficiencies in a stable population in Alaska. Aquat Mamm 36: 351–364. 10.1578/AM.36.4.2010.351.

[ref58] Logerwell EA, Schaufler LE (2005) New data on proximate composition and energy density of Steller Sea lion (*Eumetopias jubatus*) prey fills seasonal and geographic gaps in existing information. Aquat Mamm 31: 62–82. 10.1578/AM.31.1.2005.62.

[ref59] McHuron EA, Adamczak S, Arnould JP, Ashe E, Booth C, Bowen WD, Williams R (2022) Key questions in marine mammal bioenergetics. Conserv Physiol 10: coac055. 10.1093/conphys/coac055. 35949259 PMC9358695

[ref60] McPherson ML, Finger DJ, Houskeeper HF, Bell TW, Carr MH, Rogers-Bennett L, Kudela RM (2021) Large-scale shift in the structure of a kelp forest ecosystem co-occurs with an epizootic and marine heatwave. Commun Biol 298. 10.1038/s42003-021-01827-6 PMC7935997 33674760

[ref61] Metcalfe NB, Bellman J, Bize P, Blier PU, Crespel A, Dawson NJ, Monaghan P (2023) Solving the conundrum of intra-specific variation in metabolic rate: a multidisciplinary conceptual and methodological toolkit: New technical developments are opening the door to an understanding of why metabolic rate varies among individual animals of a species. Bioessays 45: 2300026. 10.1002/bies.202300026. 37042115

[ref62] Miller LK (1978) Energetics of the northern fur seal in relation to climate and food resources of the Bering Sea. University of Alaska, Institute of Arctic Biology

[ref63] Miller MA, Moriarty ME, Henkel L, Tinker MT, Burgess TL, Batac FI, Johnson CK (2020) Predators, disease, and environmental change in the nearshore ecosystem: mortality in southern sea otters (*Enhydra lutris nereis*) from 1998–2012. Frontiers Mar Sci 7: 582. 10.3389/fmars.2020.00582.

[ref64] Miller MA, Newberry CA, Sinnott DM, Batac FI, Greenwald K, Reed A, Shapiro K (2023) Newly detected, virulent toxoplasma gondii COUG strain causing fatal steatitis and toxoplasmosis in southern sea otters (*Enhydra lutris nereis*). Frontiers Mar Sci 10: 1116899. 10.3389/fmars.2023.1116899.

[ref65] Molnár PK, Klanjscek T, Derocher AE, Obbard ME, Lewis MA (2009) A body composition model to estimate mammalian energy stores and metabolic rates from body mass and body length, with application to polar bears. J Exp Biol 212: 2313–2323. 10.1242/jeb.026146. 19617423

[ref112] Morrison P, Rosenmann M, Estes JA (1974) Metabolism and thermoregulation in the sea otter. Physiological zoology 47: 218–29.

[ref66] Moxley JH, Nicholson TE, Van Houtan KS, Jorgensen SJ (2019) Non-trophic impacts from white sharks complicate population recovery for sea otters. Ecol Evol 9: 6378–6388. 10.1002/ece3.5209. 31236228 PMC6580303

[ref67] Mr̊rtensson PE, Gotaas AL, Norddy ES, Blix AS (1996) Seasonal changes in energy density of prey of Northeast Atlantic seals and whales. Mar Mamm Sci 12: 635–640. 10.1111/j.1748-7692.1996.tb00080.x.

[ref68] Nicholson TE, Mayer KA, Staedler MM, Gagné TO, Murray MJ, Young MA, Van Houtan KS (2020) Robust age estimation of southern sea otters from multiple morphometrics. Ecol Evol 10: 8592–8609. 10.1002/ece3.6493. 32884643 PMC7452773

[ref69] Nicholson TE, Mayer KA, Hazan SH, Murray MJ, Van Houtan KS, DeAngelo CM, Johnson AB, Fujii JA (2023) Advancing surrogate-rearing methods to enhance southern sea otter recovery. Biol Conserv 281: 109962. 10.1016/j.biocon.2023.109962.

[ref70] Nicholson TE, McClenachan L, Tanaka KR, Van Houtan KS (2024) Sea otter recovery buffers century-scale declines in California kelp forests. PLOS Climate 3: e0000290. 10.1371/journal.pclm.0000290.

[ref71] Noren SR, Rosen DA (2023) What are the metabolic rates of marine mammals and what factors impact this value: a review. Conserv Physiol 11: coad077. 10.1093/conphys/coad077. 37790839 PMC10545007

[ref72] Oftedal OT, Ralls K, Tinker MT, Green A (2007) Nutritional constraints on the southern sea otter in the Monterey Bay National Marine Sanctuary, and a comparison to sea otter populations at San Nicolas Island, California and Glacier, Bay, Alaska, Final report to the Monterey Bay National Marine Sanctuary and the Marine Mammal Commission.

[ref73] Osterrieder SK, Davis RW (2009) Summer foraging behaviour of Female Sea otters (*Enhydra lutris*) with pups in Simpson Bay, Alaska. Aquat Mamm 35: 481–489. 10.1578/AM.35.4.2009.481.

[ref74] Osterrieder SK, Davis RW (2011) Sea otter female and pup activity budgets, Prince William sound, Alaska. J Mar Biol Assoc UK 91: 883–892. 10.1017/S0025315410001426.

[ref75] Ostfeld RS (1982) Foraging strategies and prey switching in the California Sea otter. Oecologia 53: 170–178. 10.1007/BF00545660. 28311106

[ref76] Pagano AM, Rode KD, Atkinson SN (2017) Evaluating methods to assess the body condition of female polar bears. Ursus 28: 171–181. 10.2192/URSU-D-16-00029.1.

[ref77] Payne SF, Jameson RJ (1984) Early behavioral development of the sea otter, *Enhydra lutris*. J Mammal 65: 527–531. 10.2307/1381114.

[ref78] Pearson HC, Davis RW (2005) Behavior of territorial male sea otters (Enhydra lutris) in Prince William sound, Alaska. Aquat Mamm 31: 226–233. 10.1578/AM.31.2.2005.226.

[ref79] Pirotta E (2022) A review of bioenergetic modelling for marine mammal populations. Conserv Physiol 10: coac036. 10.1093/conphys/coac036. 35754757 PMC9215292

[ref80] Prothero J (1995) Bone and fat as a function of body weight in adult mammals. Comp Biochem Physiol A 111: 633–639. 10.1016/0300-9629(95)00050-H. 7671155

[ref81] Ralls K, Siniff DB (1990) Time budgets and activity patterns in California Sea otters. J Wildl Manage 54: 251–259. 10.2307/3809038.

[ref109] Riedman ML, Estes JA, Staedler MM, Giles AA, Carlson DR (1994) Breeding patterns and reproductive success of California sea otters. The Journal of wildlife management 1: 391–399.

[ref82] Rotterman LM, Monnett C (2002) Length-mass and total body length of adult female sea otters in Prince William sound before and after the Exxon Valdez oil spill. Mar Mamm Sci 18: 977–993. 10.1111/j.1748-7692.2002.tb01086.x.

[ref83] Ryg M, Lydersen C, Knutsen LØ, Bjørge A, Smith TG, Øritsland NA (1993) Scaling of insulation in seals and whales. J Zool 230: 193–206. 10.1111/j.1469-7998.1993.tb02682.x.

[ref84] Schmidt-Nielsen K (1997) Animal Physiology: Adaptation and Environment, 5^th^ edn. New York, NY: Cambridge University Press, 10.1017/9780511801822.

[ref85] Sherrod SK, Estes JA, White CM (1975) Depredation of sea otter pups by bald eagles at Amchitka Island, Alaska. J Mammal 56: 701–703. 10.2307/1379491.

[ref86] Silva MP, Oliveira C, Prieto R, Silva MA, New L, Pérez-Jorge S (2024) Bioenergetic modelling of a marine top predator's responses to changes in prey structure. Ecol Evol 14: e11135. 10.1002/ece3.11135. 38529024 PMC10961477

[ref87] Smith JG, Tomoleoni J, Staedler M, Lyon S, Fujii J, Tinker MT (2021) Behavioral responses across a mosaic of ecosystem states restructure a sea otter–urchin trophic cascade. Proc Nat Acad Sci 118: e2012493118. 10.1073/pnas.2012493118. 33836567 PMC7980363

[ref88] Smith JG, Malone D, Carr MH (2024) Consequences of kelp forest ecosystem shifts and predictors of persistence through multiple stressors. Proc R Soc B 291: 20232749. 10.1098/rspb.2023.2749 PMC10846955 38320605

[ref89] Sparling CE, Speakman JR, Fedak MA (2006) Seasonal variation in the metabolic rate and body composition of female grey seals: fat conservation prior to high-cost reproduction in a capital breeder? J Comp Physiol B 176: 505–512. 10.1007/s00360-006-0072-0. 16506041

[ref115] Sparling CE, Thompson D, Fedak MA, Gallon SL, Speakman JR (2008) Estimating field metabolic rates of pinnipeds: doubly labelled water gets the seal of approval Functional Ecology 22: 245–54.

[ref90] Spitz J, Ridoux V, Trites AW, Laran S, Authier M (2018) Prey consumption by cetaceans reveals the importance of energy-rich food webs in the Bay of Biscay. *Progr* Oceanogr166:148-158.

[ref91] Staedler MM (2011) Maternal care and provisioning in the southern sea otter (Enhydra lutris nereis): Reproductive consequences of diet specialization in an apex predator. University of California, Santa Cruz

[ref92] Tarasoff FJ (1974) Anatomical adaptations in the river otter, sea otter and harp seal with reference to thermal regulation. *In* R. J. Harrison (ed.). Funct Anat Mar Mammals 2: 111–141.

[ref93] Thometz NM, Tinker MT, Staedler MM, Mayer KA, Williams TM (2014) Energetic demands of immature sea otters from birth to weaning: implications for maternal costs, reproductive behavior and population-level trends. J Exp Biol 217: 2053–2061. 10.1242/jeb.099739. 24920834

[ref113] Thometz NM, Murray MJ, Williams TM (2015) Ontogeny of oxygen storage capacity and diving ability in the southern sea otter (Enhydra lutris nereis): costs and benefits of large lungs. Physiological and Biochemical Zoology 88: 311–27. 25860829 10.1086/681019

[ref94] Thometz NM, Kendall TL, Richter BP, Williams TM (2016a) The high cost of reproduction in sea otters necessitates unique physiological adaptations. J Exp Biol 219: 2260–2264. 10.1242/jeb.138891. 27489214

[ref95] Thometz NM, Staedler MM, Tomoleoni JA, Bodkin JL, Bentall GB, Tinker MT (2016b) Trade-offs between energy maximization and parental care in a central place forager, the sea otter. Behav Ecol 27: 1552–1566. 10.1093/beheco/arw089.

[ref96] Tinker MT, Bentall G, Estes JA (2008) Food limitation leads to behavioral diversification and dietary specialization in sea otters. Proc Nat Acad Sci 105: 560–565. 10.1073/pnas.0709263105. 18195370 PMC2206575

[ref97] Tinker MT, Guimarães PR Jr, Novak M, Marquitti FMD, Bodkin JL, Staedler M, Estes JA (2012) Structure and mechanism of diet specialisation: testing models of individual variation in resource use with sea otters. Ecol Lett 15: 475–483. 10.1111/j.1461-0248.2012.01760.x. 22414160

[ref98] Tinker MT, Hatfield BB, Harris MD, Ames JA (2016) Dramatic increase in sea otter mortality from white sharks in California. Mar Mamm Sci 32: 309–326. 10.1111/mms.12261.

[ref99] Tinker MT, Tomoleoni JA, Weitzman BP, Staedler M, Jessup D, Murray MJ, ... Conrad P (2019) *Southern sea otter (Enhydra lutris nereis) population biology at Big Sur and Monterey, California--Investigating the consequences of resource abundance and anthropogenic stressors for sea otter recovery* (No. 2019-1022). US Geological Survey.

[ref110] Tinker MT, Tomoleoni J, LaRoche N, Bowen L, Miles AK, Murray M, Staedler M, Randell Z (2017) Southern sea otter range expansion and habitat use in the Santa Barbara Channel, California. US Geological Survey.

[ref100] Trumble SJ, Barboza PS, Castellini MA (2003) Digestive constraints on an aquatic carnivore: effects of feeding frequency and prey composition on harbor seals. J Comp Physiol B 173: 501–509. 10.1007/s00360-003-0358-4. 12856134

[ref101] Walker KA, Davis JW, Duffield DA (2008) Activity budgets and prey consumption of sea otters (*Enhydra lutris kenyoni*) in Washington. Aquat Mamm 34: 393–401. 10.1578/AM.34.4.2008.393.

[ref102] Wilkin SM (2003) Nocturnal foraging ecology and activity budget of the sea otter (Enhydra lutris) in Elkhorn Slough, California. Doctoral dissertation, San Francisco State University.

[ref103] Williams TM (1989) Swimming by sea otters: adaptations for low energetic cost locomotion. J Comp Physiol A 164: 815–824. 10.1007/BF00616753. 2724187

[ref104] Winer JN, Liong SM, Verstraete FJM (2013) The dental pathology of southern sea otters (*Enhydra lutris nereis*). J Comp Pathol 149: 346–355. 10.1016/j.jcpa.2012.11.243. 23348015

[ref105] Wolt RC, Finerty SE, Davis RW (2014) Time and energy allocation of female sea otters (*Enhydra lutris*) with pups in Alaska. J Exp Mar Biol Ecol 461: 93–101. 10.1016/j.jembe.2014.07.015.

[ref106] Yeates LC, Williams TM, Fink TL (2007) Diving and foraging energetics of the smallest marine mammal, the sea otter (*Enhydra lutris*). J Exp Biol 210: 1960–1970. 10.1242/jeb.02767. 17515421

[ref107] Yee JL, Tomoleoni JA, Kenner MC, Fujii J, Bentall GB, Tinker MT, Hatfield BB (2020) Southern (California) sea otter population status and trends at San Nicolas Island, 2017–2020 No. 2020-1115. US Geological Survey.

[ref108] Yee JL, Tomoleoni JA, Kenner MC, Fujii JA, Bentall GB, Staedler MM, Hatfield BB (2023) Southern (California) sea otter population status and trends at San Nicolas Island, 2020–2023 No. 2023-1071. US Geological Survey.

